# The development of a novel zeolite-based assay for efficient and deep plasma proteomic profiling

**DOI:** 10.1186/s12951-024-02404-9

**Published:** 2024-04-10

**Authors:** Nan Li, Jingnan Huang, Shangwen He, Qiaocong Zheng, Feng Ye, Zhengxing Qin, Dong Wang, Ting Xiao, Mengyuan Mao, Zhenhua Zhou, Tingxi Tang, Longshan Zhang, Xiaoqing Wang, Yingqiao Wang, Ying Lyu, Laiyu Liu, Lingyun Dai, Jigang Wang, Jian Guan

**Affiliations:** 1 Department of Radiation Oncology, Nanfang Hospital, Southern Medical University, Guangzhou, 510515 Guangdong China; 2 Department of Nephrology, Shenzhen Key Laboratory of Kidney Diseases, Shenzhen People’s Hospital, (The Second Clinical Medical College, Jinan University, The First Affiliated Hospital, Southern University of Science and Technology), Shenzhen, 518020 Guangdong China; 3 School of Medicine, Southern University of Science and Technology, Shenzhen, 518055 Guangdong China; 4 Department of Oncology, People’s Hospital of YangJiang, Yangjiang, 529500 Guangdong China; 5 State Key Laboratory of Heavy Oil Processing, College of Chemical Engineering, China University of Petroleum (East China), Qingdao, 266580 Shandong China; 6 College of Chemistry and Chemical Engineering, China University of Petroleum (East China), Qingdao, 266580 Shandong China; 7 Department of Traditional Chinese Medicine, Nanfang Hospital,, Southern Medical University, Guangzhou, 510515 Guangdong China; 8 Chronic Airways Diseases Laboratory, Department of Respiratory and Critical Care Medicine, Nanfang Hospital, Southern Medical University, Guangzhou, 510515 Guangdong China; 9 Guangdong Province Key Laboratory of Molecular Tumor Pathology, Guangzhou, 510515 Guangdong China

**Keywords:** Plasma, Proteomics, Zeolite, Protein corona

## Abstract

Plasma proteins are considered the most informative source of biomarkers for disease diagnosis and monitoring. Mass spectrometry (MS)-based proteomics has been applied to identify biomarkers in plasma, but the complexity of the plasma proteome and the extremely large dynamic range of protein abundances in plasma make the clinical application of plasma proteomics highly challenging. We designed and synthesized zeolite-based nanoparticles to deplete high-abundance plasma proteins. The resulting novel plasma proteomic assay can measure approximately 3000 plasma proteins in a 45 min chromatographic gradient. Compared to those in neat and depleted plasma, the plasma proteins identified by our assay exhibited distinct biological profiles, as validated in several public datasets. A pilot investigation of the proteomic profile of a hepatocellular carcinoma (HCC) cohort identified 15 promising protein features, highlighting the diagnostic value of the plasma proteome in distinguishing individuals with and without HCC. Furthermore, this assay can be easily integrated with all current downstream protein profiling methods and potentially extended to other biofluids. In conclusion, we established a robust and efficient plasma proteomic assay with unprecedented identification depth, paving the way for the translation of plasma proteomics into clinical applications.

**Graphical Abstract:**

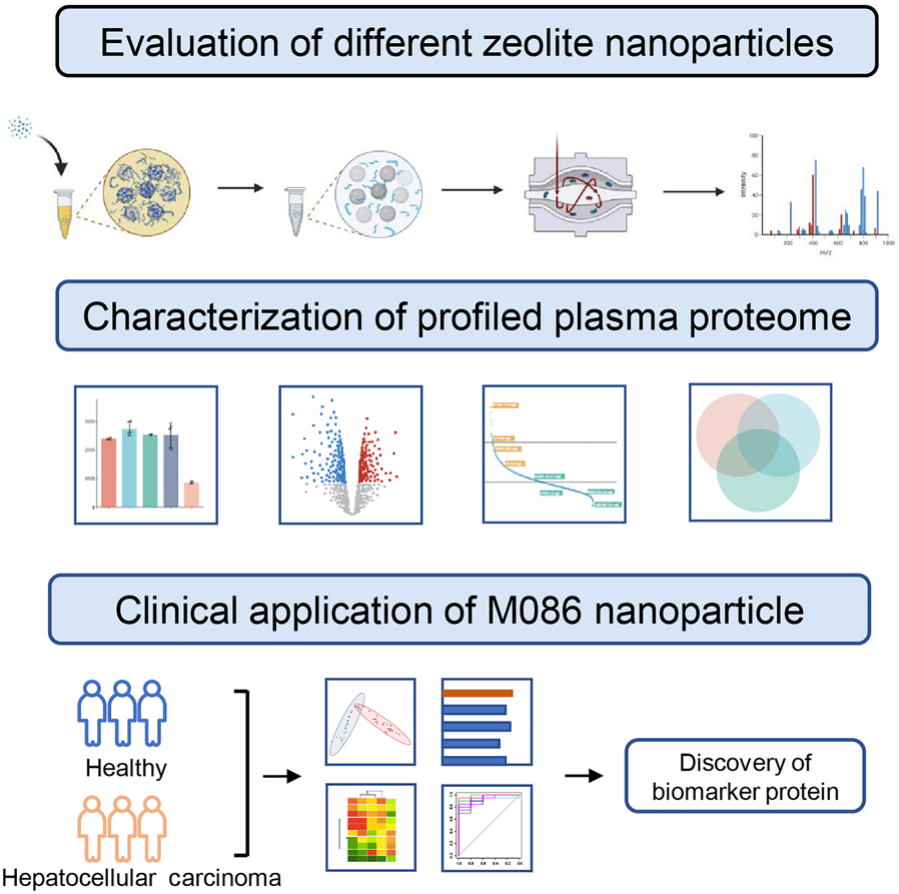

**Supplementary Information:**

The online version contains supplementary material available at 10.1186/s12951-024-02404-9.

## Introduction

The circulating proteins in the plasma, serum, and whole blood are among the most promising biomarkers for clinical diagnosis: they reflect the health status of individuals and exhibit great potential for disease diagnosis and monitoring [1, 2]. However, the large-scale application of plasma proteomics remains highly challenging, mainly due to the enormous complexity of the plasma proteome and is vast dynamic range of protein abundances [3, 4]. Currently, plasma proteomics methods typically depend on immunoaffinity-based strategies to deplete high-abundance proteins. However, this strategy is too expensive for large-scale studies [5–7].

Protein–nanoparticle (NP) interaction was first studied in the early 1950s [8, 9], when scientists discovered that a thin layer of protein, known as the “protein corona”, formed on the surface of NPs once mixed with biological fluids (e.g., plasma, serum, and urine) [10]. However, the exact molecular mechanisms underlying this phenomenon have not been elucidated. In recent years, it has become increasingly clear that the formation and composition of protein coronas are primarily influenced by the physicochemical properties of NPs [9, 11–13] and proteins [14–17]. In turn, the surface adsorption of proteins strongly affects the biological characterization and function of NPs, which play critical roles in targeted drug delivery [18–20], non-invasive biosensors [21, 22], photothermal therapy [23, 24] and clinical diagnostics [25, 26].

In light of the progression in understanding protein coronas, many researchers have started to explore the application of nanomaterials in plasma proteomics by modifying their physical, chemical and biological characteristics [9, 11–13, 27–30]. Blume et al. utilized a panel of five NPs with different physicochemical properties and protein-binding affinities to decrease the dynamic range of protein abundances and quantified an average of 1664 proteins from the plasma of a non-small-cell lung cancer (NSCLC) cohort in data-independent acquisition (DIA) mode [29]. Ferdosi et al. [30] managed to increase the number of identified proteins to ~ 3000 with optimizing data acquisition conditions and search engines, suggesting a possible approach to designing a multiple-NP panel for a more comprehensive view of the plasma proteome [30].

Zeolites are inorganic microporous aluminosilicates that are widely used in catalysis, separation, and sorption processes [31–33]. Due to their high stability, nontoxicity and tunability, zeolite materials have been incorporated into many advanced applications in the medical, food, and cosmetic industries [32, 34, 35]. Notably, NaY, a faujasite (FAU)-type zeolite, was found to have low binding affinity for serum albumin and haemoglobin, which are the two most abundant plasma proteins [33, 36]. Thus, we hypothesized that the application of NaY might reduce the dynamic range of the plasma proteome and enable the construction of a novel plasma protein detection assay.

**Fig. 1:**
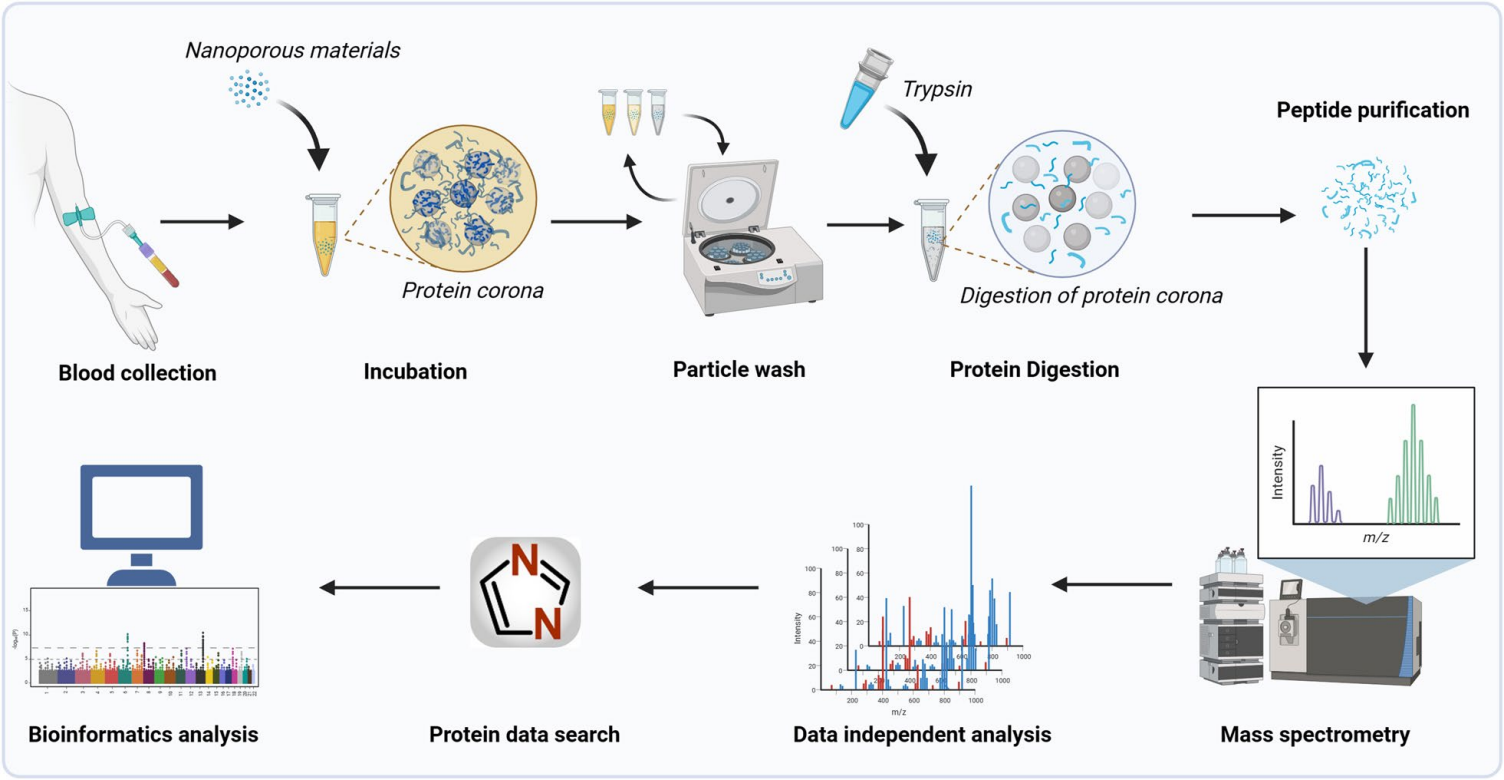
Schematic diagram of the workflow. The zeolite-based plasma proteomic profiling workflow consisted of 7 main steps: (1) blood collection; (2) incubation of different nanoparticles (NPs) with plasma to form a protein corona; (3) washing and cleaning of NPs; (4) on-bead protein digestion; (5) peptide purification; (6) data processing; and (7) bioinformatics analysis

In this study, we evaluated the performance of the zeolite NaY and similar materials with different physicochemical properties in the interrogation of the plasma proteome and compared the results to those obtained using neat and depleted plasma (Fig. 1). In addition, we tested the effectiveness of NaY-based plasma proteomic assay for distinguishing hepatitis B virus-related hepatocellular carcinoma (HCC) patients from the age- and sex-matched healthy controls.

## Results

### Preparation and characterization of zeolite-based nanoparticles (NPs)

Given that NP–protein adsorption is highly selective and is influenced by their own physicochemical properties of both the proteins and the NPs [9, 11, 12, 28, 32, 33], we first designed and synthesized a panel of zeolite-based NPs with different physicochemical characteristics, including M086 (NaY, Si/Al = 2.6), M158 (CHA, Si/Al = 2.0) and M909 (FAU, Si/Al = 2.0) (Additional file 6: Table S1). We then evaluated the adsorption capacity of these NPs and relationship between concentration of NPs for plasma proteins (Additional file 1: Fig S1A) by SDS‒PAGE [37]. Our results showed that all three NPs reduced the dynamic range of plasma proteins and different concentration of NPs hardly changed the pattern of absorbed protein, especially above 0.5 mg ~ 200 mL. (Additional file 1: Fig. S1B).

**Fig. 2:**
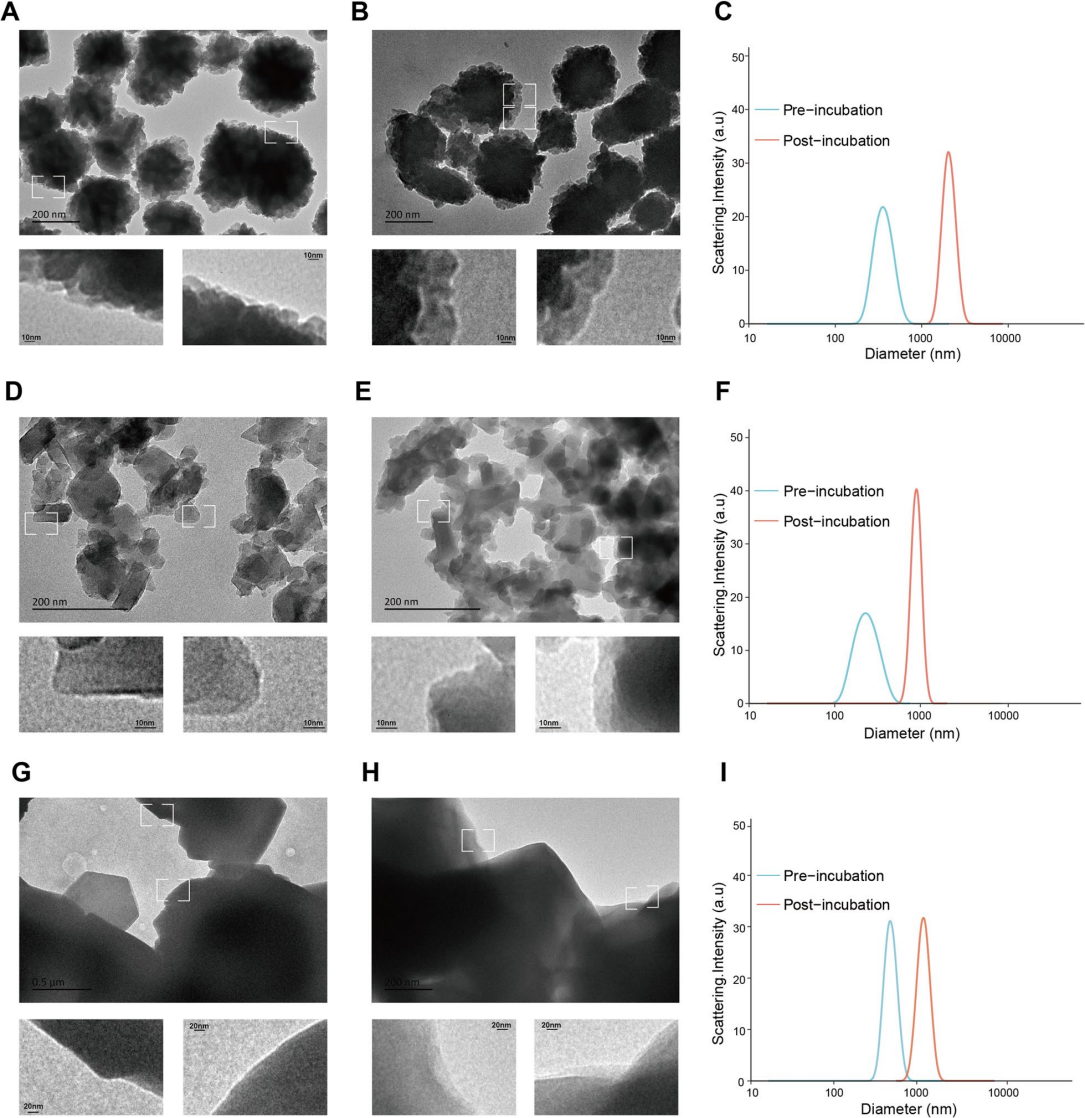
Characteristics of the three zeolite-based NPs before and after incubation with plasma proteins.** A**–**C**: M158; **D**–**F**: M909; **G**–**H**: M086. **A**, **B**, **D**, **E**, **G**, and **H** show TEM images of the surfaces of M158 (A: pre-incubation; B: post-incubation), M909 (D: pre-incubation; E: post-incubation), and M086 (G: pre-incubation; H: post-incubation), respectively. **B**, **E** and **H:** The average DLS results of three replicates of each material. (Blue line: pre-incubation NP; red line: post-incubation)

The size and morphology of these NPs were then characterized by dynamic light scattering (DLS) and transmission electron microscopy (TEM) (Fig. 2 and Appendix 1). The result revealed that the mean particle sizes of M158, M909 and M086 were 370.7 ± 25.8 nm (Fig. 2A and C), 244.3 ± 29.3 nm (Fig. 2D and F) and 488.6 ± 13.4 nm, respectively (Fig. 2G and I). After incubation with plasma, the diameters of the three types of NPs significantly increased to 2086.7 ± 57.7 nm (Fig. 2B and C), 899.1 ± 7.4 nm (Fig. 2E and F) and 1216.1 ± 31.2 nm (Fig. 2H and I). The increase in hydrodynamic size after incubation with plasma could be visualized by the faint halos that formed on the surface of all three NPs, which is consistent with previously reported results [38–40] (Fig. 2). The above results confirmed that all three zeolite-based NPs can form protein coronas and reduce the relative quantity of high-abundance proteins.

### The interrogation of the plasma proteome using the three zeolite-based NPs

Next, we evaluated the performance of the three zeolite-based NPs by interrogating the plasma proteome by mass spectrometric analysis and compared the results with those obtained using the commercially available High-Select^™^ Top14 Abundant Protein Depletion Resin (depleted). Untreated, neat plasma (neat) was also characterized under the same conditions as a reference. The key parameters included the depth of the measured proteome (number of protein groups and peptides), the quantification accuracy (coefficient of variation of measurements of three replicate samples) and the ease of operability (total assay time and operational simplicity).

**Fig. 3:**
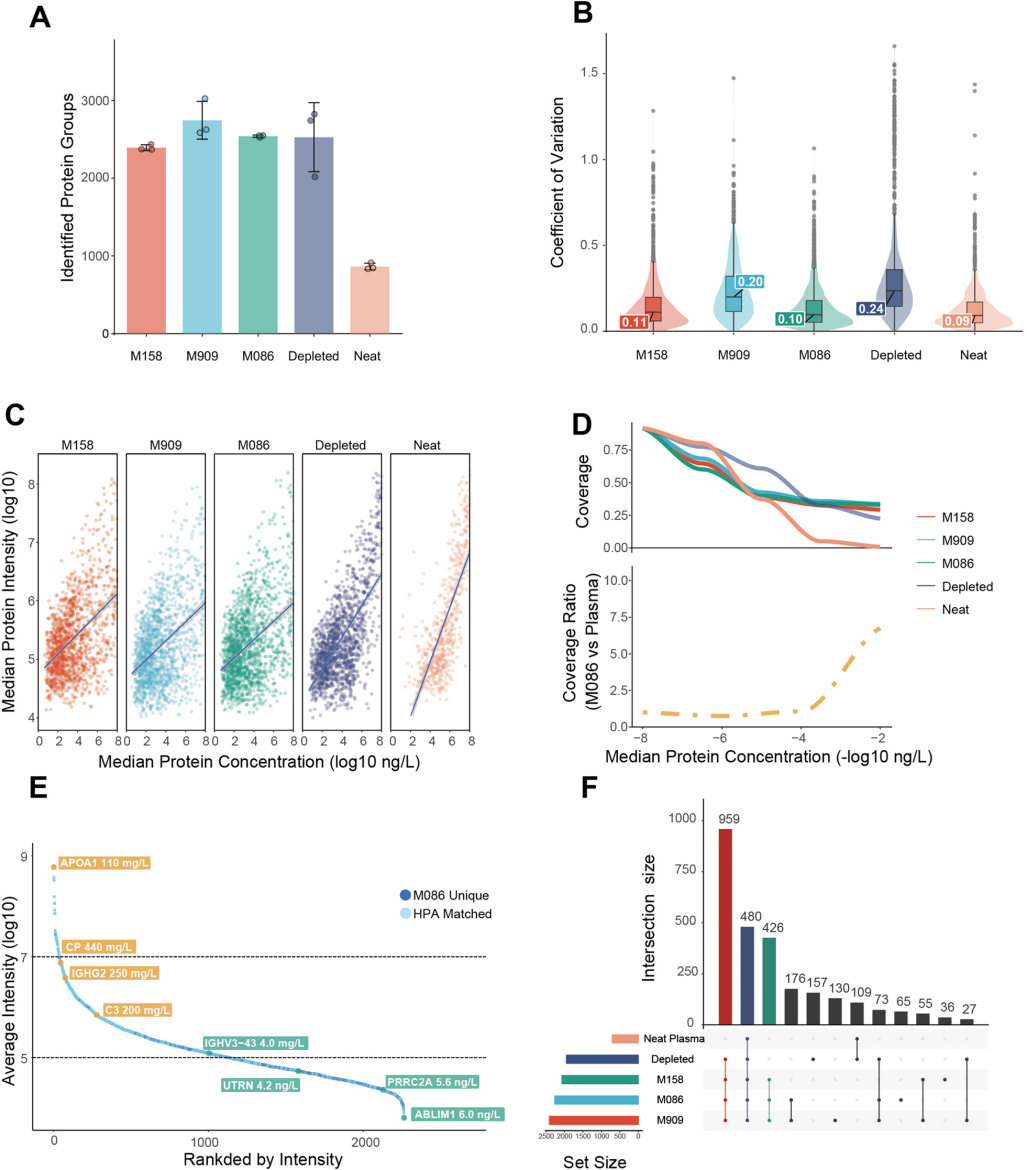
Proteomic characterization of three different NPs, depleted plasma and neat plasma. **A** The numbers of protein groups measured via five different plasma proteomic methods, namely, in plasma treated with the three NPs, depleted plasma and neat plasma, as determined by LC‒MS/MS and DIA-NN (version 1.8.1, 1% protein and peptide FDR). M158/M909/M086: protein groups in the samples incubated with the corresponding NPs. Depleted: the protein group in plasma depleted by Thermo Fisher Scientific High-Select™ Top14 Abundant Protein Depletion Resin. Neat Plasma: the protein group of untreated plasma samples. **B** Coefficients of variation (CVs) of the proteins quantified by the five different methods. Inner boxplots represent the 25% (lower hinge), 50%, and 75% (upper hinge) quantiles. Whiskers indicate observations at or outside the hinge ± 1.5 * interquartile range (IQR). **C** Correlation of the median of the measured protein intensities with the published protein concentration in the HPA database. The blue lines are linear regression lines, and the grey shaded regions represent the 95% confidence intervals. **D** Percent coverage of the HPA database for each method (top) and relative coverage of the plasma protein database by the M086-based NP method compared to the neat plasma method (bottom) over negative log10 protein intensities. Only protein groups with complete measurements were kept. All proteins and peptides were filtered with a 1% protein and peptide false discovery rate (FDR). **E** Rank distribution of the protein groups identified by M086. Ranks by intensity and log10 intensity are shown on the X and Y axes, respectively. Light blue circles represent proteins in the HPA plasma protein database, and dark blue circles represent proteins that were uniquely identified via the M086 method. **F** UpSet plot showing the overlap of the measured protein groups between the different methods. Only protein groups with complete measurements were kept

Mass spectrometry data were acquired in data-independent acquisition (DIA) mode using a 45 min effective chromatographic gradient and processed by DIA-NN software (version 1.8.0). A total of 2732, 3099 and 2822 protein groups (detected in any of three replicates) were obtained for M158, M909 and M086, respectively (Fig. 3A). M909 exhibited a slight but not statistically significant superiority in performance among the three types of NPs. In contrast, 3006 and 1068 protein groups were identified in the depleted and neat plasma, respectively. The numbers of peptides identified in different groups are shown in Additional file  2: Fig. S2A. In terms of quantification accuracy, the median coefficient of variation (CV) of the measured protein abundances from the three types of NPs were 11%, 20% and 10% (Fig. 3B), respectively, compared to 24% and 9% for depleted and neat plasma. Notably, the number of proteins with CV less than 20% in M158 and M086 exceeded 1900, which is ten fold greater than obtained by Geyer et al. [41] and three fold greater than that obtained by Blume et al. [29]. Similar levels of accuracy were observed at the peptide level (Additional file  2: Fig. S2B–C).

To evaluate the profiling coverage and depth of the different methods, we correlated the measured protein abundances with the plasma protein concentration data from the Human Protein Atlas (HPA) database. Analysis revealed that the Pearson correlation coefficients of M158, M909, and M086 were all about 0.40, while those of depleted and neat plasma were 0.64 and 0.78, respectively (Fig. 3C). These findings are consistent with those of Blume et al. [29]. To examine how each method covers the HPA database, we calculated the coverage rate of the measured plasma proteins at different concentrations. We noted a steep decrease in the neat plasma sample (Fig. 3D). Taking M086 as an example, compared to the analysis of neat plasma, the NP method yielded much better coverage (up to ~ seven fold) of the least abundant proteins across the lowest two orders of magnitude (Fig. 3D). As shown in Fig. 3E, the intensity of some of the least abundant plasma proteins in the HPA database ranked in the middle of all proteins detected by M086. Moreover, many novel plasma proteins were identified in the lower part of the curve. These findings indicated that our assay may lead to the discovery of previously uncharacterized low-abundance plasma proteins, paving the way for the development of novel clinically relevant biomarkers.

Extracellular vehicles (EVs) are important components of plasma. They may increase the number of proteins identified but also potentially create undesired variability. To address this problem, we used the 100 most common EV proteins (top 100 from the Exocarta database) as characteristic proteins of EVs and examined their intersection with the proteins we identified. We found that 86–87 characteristic proteins of EVs were detected in the plasma treated with the different materials. Interestingly, 66 characteristic proteins were also quantitatively detected in the neat plasma (Additional file 2: Fig. S2D). Although it cannot be conclusively determined whether these proteins are exclusively derived from EVs, it is undeniable that EVs might contribute to the number of proteins we identified. Therefore, a deconvolution model using the top 100 EV protein markers from Exocarta was developed to construct an EV signature [42]. We found that the contribution of EVs was low (less than 10%) for both M086 and M158 and that the proportions in the biological replicates were stable, which further suggested that our materials were able to overcome the undesired variability introduced by plasma EVs (Additional file 2: Fig. S2E).

Long sample preparation and cleaning times have hindered the application of plasma proteomics. The analysis of the time consumption in each workflow revealed that the time spent in the washing step differed greatly for the different NPs: 1.5 h (M158), 1.8 h (M909), and 0.75 h (M086), with M086 having the shortest sample preparation workflow (Additional file 6: Table S2). This difference is likely due to the different mechanical and physical properties of the three types of NPs, which affect the separation and resuspension processes.

In summary, all three types of NPs markedly increased the depth and coverage of the measured proteome compared to those of the proteomes measured in neat or depleted plasma. As expected, the unique physicochemical characteristics of each NP resulted in distinct proteomic profiles (Fig. 3F, Additional file 2: Fig. S2F).

### Characterization of the plasma proteome recovered by M086

**Fig. 4:**
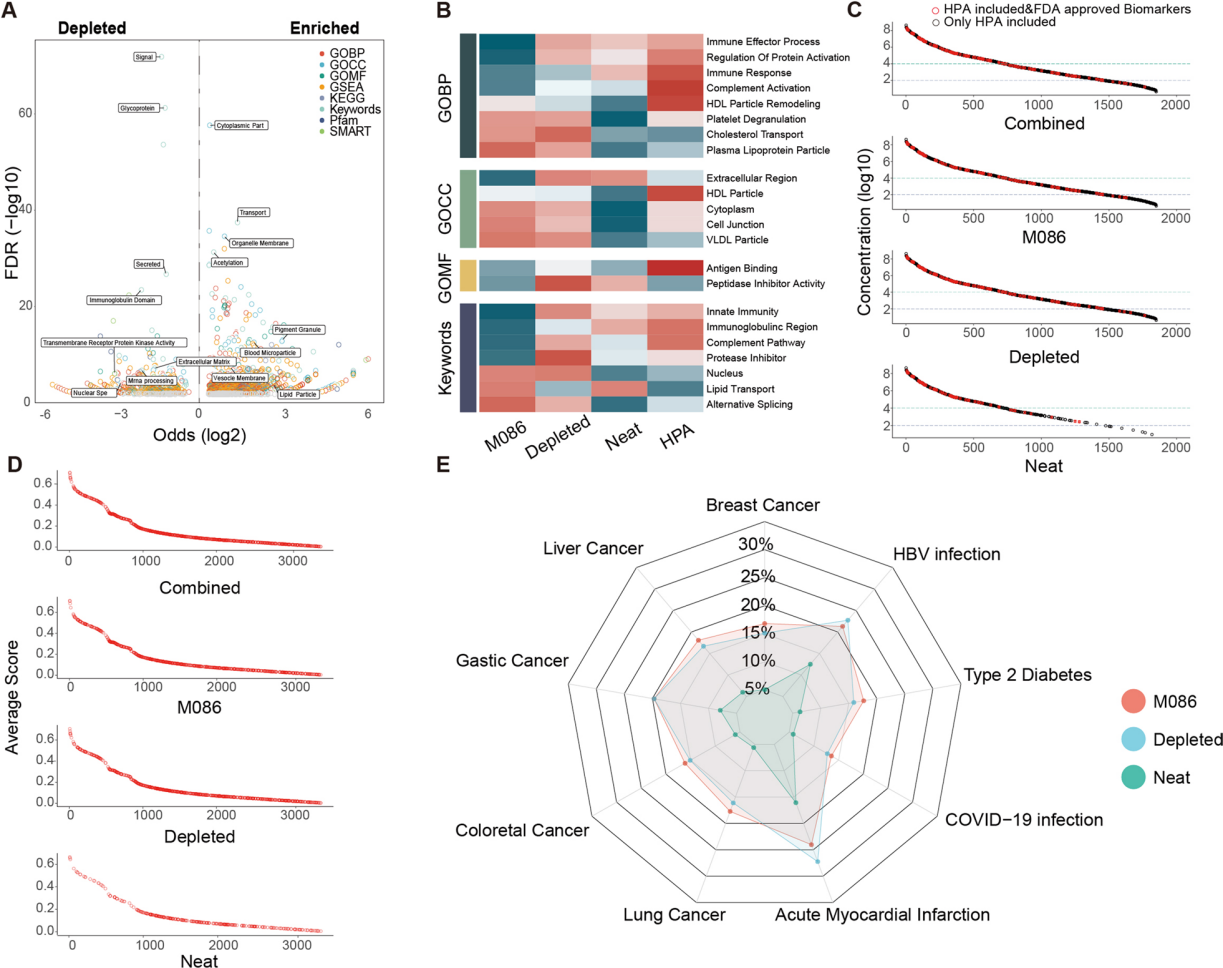
Characterization of the plasma proteome recovered by M086. **A** Volcano plot showing the annotation enrichment analysis for functional pathways (GOCC, GOBP, GOMF, KEGG, Uniports Keywords, Pfam, and SMART) of proteins detected by using M086 compared to those in the HPA database. **B** 1D annotation enrichment analysis comparing the protein intensity distribution (median intensity across assay triplicates, requiring three out of three quantifications) of each method to the average of all methods. **C** Matching of M086-recovered, depleted plasma and neat plasma samples to the HPA included & FDA approved biomarkers. Ranked intensities for all proteins detected by at least one method are shown in the top panel (combined). **D** Comparison of M086-recovered, depleted plasma and neat plasma samples with the Open Targets database. The rank average score for all proteins detected by at least one method is shown in the top panel (combined). **E** The radar plot shows the performance of M086-recovered, depleted plasma and neat plasma analysis in identifying tumours (breast, liver, gastric, colorectal and lung cancer), chronic diseases (type 2 diabetes and HBV infection) and acute diseases (COVID-19 infection and acute myocardial infarction).

Given the balanced physiochemical properties and performance of M086, in subsequent studies, we focused on characterizing the proteome recovered via the M086 method. With reference to the HPA database, we first performed enrichment analysis for the detected plasma proteins (enriched) and non-detected plasma proteins (depleted) via the M086 method on the basis of several different databases, including the Gene Ontology Cell Component (GOCC), Molecular Function (GOMF), and Biological Process (GOBP) databases, the Kyoto Encyclopedia of Genes and Genomes (KEGG), the UniProt Keywords database, the protein families database (Pfam), and the Simple Modular Architecture Research Tool database (SMART). The results suggested that M086 preferentially enriched proteins with keywords including “Cytoplasmic part”, “Organelle membrane”, “Lipid particle” and “Acetylation”, whereas proteins involved in “Secretory”, “Glycoprotein” and “Immunoglobulin Domain” were relatively underrepresented (Fig. 4A, Additional file  3: Fig. S3).

Subsequently, we conducted a 1D annotation enrichment analysis [43] to further investigate the differences in the levels of the measured proteins in M086 plasma samples compared to those in depleted and neat plasma samples (Fig. 4B). In terms of GOBP, we revealed that proteins adsorbed by M086 were enriched in “Plasma Lipoprotein Particle Assembly”, “Platelet Degranulation” and “Cholesterol Transport”, while the proteins in depleted or neat plasma methods were involved mainly in signalling pathways such as “Immune Effector Process” and “Complement Activatio” GOCC annotation suggested that M086 may preferentially bind to low-density lipoproteins and intracellular vesicle proteins. M086 also significantly enriched proteins involved in “Lipid transport”, “Alternative splicing” and “Nucleus” but showed relative depletion of “Immunoglobulin region” and “Complement pathway”.

Next, we mapped the FDA-approved protein biomarkers to the list of proteins measured using M086 and by other methods (Fig. 4C). Our results suggested that the M086 method dramatically increased the total number of FDA-approved biomarkers and displayed greater sensitivity for low-abundance biomarkers than traditional methods (Fig. 4D). To assess the ability of M086 to discover potential new biomarkers, we selected the Open Targets database as a benchmark dataset and found that the coverage of potential targets by M086 was significantly higher than that of neat plasma and as similarly high level to depleted plasma (Fig. 4E). This finding also reflects the clinical value of M086-based plasma proteomics for the potential discovery of new biological targets.

Overall, M086 exhibited a distinct enrichment profile in the plasma proteome and showed greater coverage and depth for both known and unknown targets, providing a powerful option for the comprehensive interrogation of plasma proteomics.

### Application of M086-based plasma proteomics profiling to an HCC cohort

To demonstrate the performance of the M086-based plasma proteomic assay for identifying biomarkers in a disease, we retrospectively collected 27 patients with hepatitis B-related hepatocellular carcinoma (HCC) at different Barcelona Clinic Liver Cancer (BCLC) stages and 25 age- and sex-matched healthy individuals as controls (Additional file 4: Fig. S4A-B, Additional file 6: Table S4 and S5). For better modelling in large cohort applications, we decided to use a 45 min gradient strategy, which balances proteome depth and quantification throughput.

**Fig. 5:**
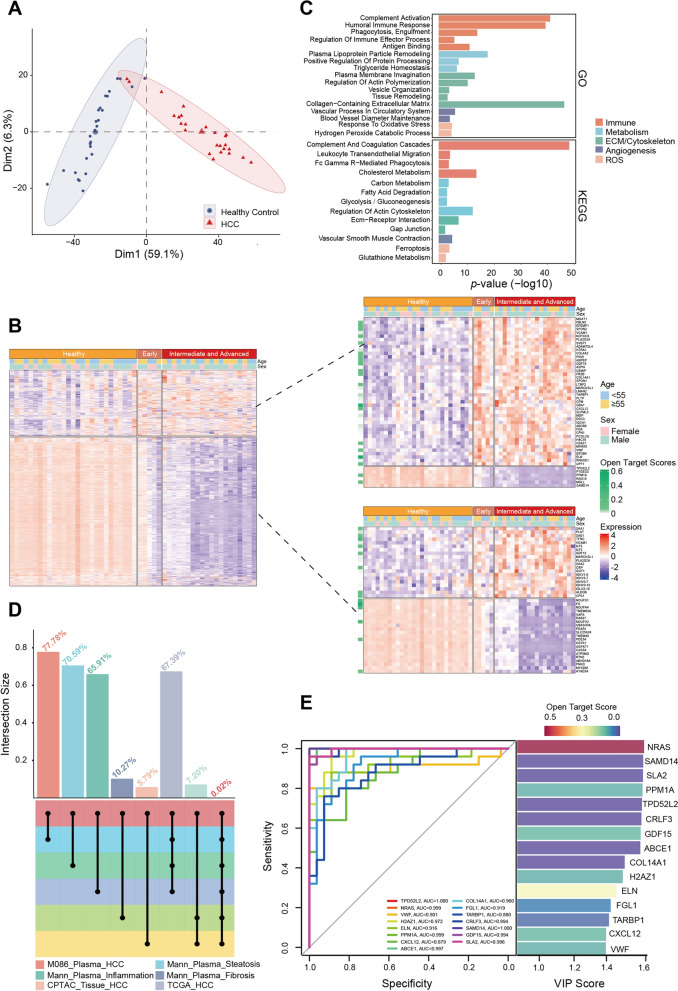
Application of M086-based plasma proteomics in an HCC cohort. **A** Principal component analysis (PCA) showing significant differences between the healthy population group (blue) and the HCC patient group (red). **B** Heatmap showing that healthy individuals (orange), patients with early-stage HCC (brownish yellow) and patients with intermediate-stage to advanced HCC (red) have different proteomic characteristics. The heatmap on the top right shows the top 50 differentially expressed proteins (ranked by adjusted *P* values), and the heatmap on the bottom right shows the proteins that showed a consistent trend among the three groups. All protein expression values were transformed by the Z score. **C** GO and KEGG enrichment results of the differentially expressed genes between patients with HCC and healthy individuals. **D** UpSet plot showing the overlap between biomarkers detected by M086 and those detected in previous studies. **E** The left panel shows the performance of 15 selected markers in HCC diagnosis; the right panel shows the VIP scores and the Open Targets scores for 15 selected markers

As shown by principal component analysis (PCA) and partial least squares discriminant analysis (PLS-DA), the HCC patients were well separated from healthy individuals (Fig. 5A, Additional file 4: Fig. S4C-D). We noted that two HCC samples classified among the healthy population were early-stage (BCLC-A stage) samples. Subsequently, we divided the cohorts into a healthy group, an early-stage (BCLC-A stage) group and an intermediate-stage and advanced-stage (BCLC-B/C stage) group (Fig. 5B, left) to investigate the differentially expressed proteins among them (Fig. 5B, upper right). The expression of several differentially expressed proteins exhibited consistent patterns during different stages of HCC progression, suggesting that these proteins may be involved in HCC progression (Fig. 5B, bottom right). KEGG analysis revealed that the differentially expressed proteins were enriched in immune- and metabolism-related pathways (Fig. 5C).

Furthermore, we compared our findings with several previous studies that examined biomarkers for hepatitis (Mann_Plasma_Inflammation), cirrhosis (Mann_Plasma_Fibrosis), HBV-related HCC (CPTAC_Tissue_HCC), and liver cancer (TCGA_HCC) [44, 45] (Fig. 5D). Despite vast differences in cohorts and methods, we observed an overlap of more than 60% (24/34 in hepatitis; 29/44 in fibrosis) in the disease biomarkers. Moreover, the plasma proteome of HCC (M086_Plasma_HCC) has been shown to be moderately correlated with that of a tissue proteomic dataset (CPTAC_Tissue_HCC), with more than 200 overlapping proteins [45].

One of main clinical application of plasma proteomics is disease diagnosis; therefore, we asked whether the novel plasma protein assay could generate biomarker proteins to differentiate HCC patients from healthy individuals. The differentially expressed proteins (DEPs) were screened using the R package “edgeR” (*P* value < 0.05 and |log2FC|> 1), orthogonal partial least-squares discrimination analysis (PLS-DA; VIP > 1) and receiver operating characteristic curve (ROC; AUC > 0.7). A total of 481 candidate proteins were obtained by taking the intersection of three groups of DEPs. To reduce the noise and probability of overfitting, we performed LASSO regression on the target proteins, and 15 proteins were retained for subsequent model construction (Additional file 5: Fig. S5A-B). The AUCs of these candidate proteins reached 0.88, with TPD52L2 and SAMD14 reaching 1.0. Subsequently, we ranked the 15 proteins by VIP score and annotated them with the importance score in HCC from the Open Targets database. Among them, vWF is the target of the FDA-approved antiplatelet drug ARC-1779. Several studies have reported that vWF may play an important role in the progression of liver disease [46, 47] and anti-vWF treatment is beneficial for preventing brain metastases [48].

As the early diagnosis of tumours remains challenging, we also investigated whether M086-based plasma proteomics could distinguish patients with early-stage disease (stage A) and patients with intermediate and advanced-stage HCC. Surprisingly, the results indicated that the AUCs of several proteins reached 0.9 (Additional file 5: Fig. S5C). Among them, NRAS, ABCE1, CRLF3 and SLA2 were significantly associated with HCC stage (Additional file 5: Fig. S5D).

Since plasma samples are susceptible to contamination by platelets and erythrocytes [42], we examined the overlap between the 15 selected characteristic proteins and the platelet and erythrocyte proteome signature proteins published by Geyer et al. Neither platelet nor erythrocyte signature proteins were found among them, which further validates that our protein signatures are not related to common contamination or disruptions.

## Discussion

The major challenge of plasma proteomics is the limited proteome coverage, which restricts its application in clinical settings. To increase proteome depth, strategies such as extensive peptide fractionation, isobaric labelling and pooling have been adopted at the expense of reduced throughput and increased cost [30, 49]. Here, inspired by the selective binding of proteins by zeolites, we developed a plasma proteomic assay based on a single nanoporous material M086, which is capable of detecting approximately 3000 proteins spanning more than 7 orders of magnitude with a median CV of 10% using a 45 min gradient. M086 cells exhibited improved tolerance to interference from immune-related proteins, including complement proteins and immunoglobulins, which are the most abundant plasma proteins. Compared with the conventional immunoaffinity depletion method, M086 is more cost effective and stable and is therefore suitable for large-scale clinical studies (Additional file 6: Table S3). Using an M086-based proteomic assay, more than 180 FDA-approved biomarkers were identified in plasma, suggesting new possibilities for translational research. In our retrospective investigation of the proteomic profiles of 52 HBV-associated HCC patients and age- and sex-matched healthy controls using M086, over 200 biomarkers were identified in the HCC plasma and tissue proteome (CPTAC database) [45] and, to a lesser extent, in the HCC tissue transcriptome (TCGA database), suggesting that the M086-based proteomic assay may serve as a reliable, practical tool for studies of human diseases.

A combination of ROC analysis, PLS-DA and LASSO regression was used to screen potential targets, resulting in a 15-protein signature. vWF [48, 50, 51], GDF15 [52, 53], CXCL12 [54, 55], COL14A1 [56], SAMD14 [57], PPM1A [58, 59] and TPD52L2 [60, 61] have been reported to be associated with HCC carcinogenesis and progression. Among them, vWF, a glycoprotein involved in haemostasis, is also an FDA-approved biological target for anticoagulant drugs. Some studies have also demonstrated that the inhibition of vWF can reduce tumour metastasis [50, 51].

Since early diagnosis of malignancy is difficult, we also explored the expression patterns of 15 HCC proteins in patients with early-stage HCC (BCLC stage A), patients with intermediate and to advanced-stage HCC (BCLC stage B/ C) and healthy individuals. A clear trend could be observed across different HCC stages, as the expression levels of NRAS, PPM1A, CRLF3, SLA2 and SMAD14 decreased gradually with the progression of HCC. In contrast, FGL1 and GDF15 increased with the advancement of disease, highlighting the importance of these biomarkers in the progression of HCC. However, due to the limited sample size, future large-scale studies are needed to validate these results.

One of the main features of M086 is its high degree of compatibility. M086 can be seamlessly integrated into existing mass spectrometry acquisition strategies, such as sequential window acquisition of all theoretical fragment-ion spectra (SWATH) or parallel accumulation-serial fragmentation (PASEF), providing a simple yet efficient solution to increase proteome depth. We also expect M086 to be amenable to further modifications. For example, the magnetization of M086 may help to construct a fully automated MS workflow for large-scale research. Surface modifications may affect the composition of the protein corona, leading to the increased identification of specific classes of proteins. Finally, other biological fluids, such as urine, bronchoalveolar lavage fluid and cerebrospinal fluid, could also be evaluated by M086-based proteomic analysis.

In conclusion, we established a robust and efficient plasma proteomic assay with unprecedented identification depth. Our novel plasma proteomics assay requires only one material and can be easily integrated into existing mass spectrometric workflows, striking a balance between proteome coverage, throughput and cost and presenting a new strategy to address current challenges in plasma proteomics.

## Materials and methods

### Preparation of NPs

M158, a CHA zeolite with a Si/Al ratio of 2.0 and a particle size distribution between 200 and 500 nm (Fig. 2, Additional file 6: Table S1), was prepared according to the recipe for the preparation of “K04-7”, as reported in Ghojavand et al. [62]. M909, a nanosized FAU-Y-type zeolite with a Si/Al ratio of 2.0 and a particle size smaller than 200 nm, was prepared from a precursor suspension with the following chemical composition: 8 Na_2_O: 0.7 Al_2_O_3_: 10 SiO_2_: 160 H_2_O. Following the original recipe [63], the reactants were divided to prepare two initial solutions, denoted A and B. Solution A was prepared by dissolving 2 g of NaOH (99 wt%, Sinopharm Chemical Reagent Co.) in 4 g of deionized water followed by the slow addition of 0.189 g of aluminium powder (325 mesh, 99.5%, Macklin). Solution B was prepared by mixing 10 g of colloidal silica (Ludox-HS 30, 30 wt% SiO_2_, pH=9.8; Sigma‒Aldrich) with 1.6 g of NaOH and 3.4 g of deionized H_2_O. Then, solution A was added dropwise under vigorous stirring to solution B, which was kept on ice. The resulting clear suspension was kept at room temperature for 24 h for ageing. Hydrothermal crystallization was performed at 60 °C for 24 h, after which the sample was centrifuged, washed thoroughly with deionized water, and dried at room temperature for more than 24 h before use. M086 is a commercial NaY zeolite sample with a Si/Al ratio of 2.6 and a wide crystal size distribution ranging from 500 to 2000 nm (Fig. 2). This sample was custom prepared by Nanjing/Jiangsu XFNANO Materials Tech Co., Ltd.

### Characterization of the physicochemical properties of the NPs

Dynamic light scattering (DLS) was performed on a Zetasizer Nano ZS (Malvern Instruments, Worcestershire, UK). NPs were suspended at 10 mg/mL in water with 10 min of water bath sonication prior to testing. The samples were then diluted to ~ 0.02 wt% for DLS measurements in the respective buffers. DLS was performed in water at 25 °C in disposable polystyrene semi-microcuvettes (VWR, Randor, PA, USA) with a 1 min temperature equilibration time, and the average was taken from three runs of 1 min with a 633 nm laser in 173 °C backscatter mode. The DLS results were analysed using the cumulant method.

Aqueous dispersions of NPs were prepared at a concentration of 10 mg/mL from weighted NP powders redispersed in DI water by 10 min of sonication. Then, the samples were diluted 4× with methanol (Fisher) to create a dispersion in water–methanol that was directly used for electron microscopy. The TEM grids were prepared by drop-casting 2 µL of the NP dispersion in a water–methanol mixture (25–75 v/v%) at a final concentration of 0.25 mg/mL and drying in a vacuum desiccator for approximately 24 h prior to TEM analysis. A JEOL JEM-1400 transmission electron microscope (TEM) with an accelerating voltage of 120 kV was used for the TEM measurements.

### SDS‒PAGE electrophoresis

The adsorption capacities of the different NPs were determined via SDS‒PAGE. Briefly, NPs were incubated with 20 mL of plasma and washed three times before being resuspended in SDS–PAGE loading buffer. The NPs were then boiled at 95 °C for 5 min and centrifuged at 14,000g for 5 min. The supernatants of the samples were loaded into a 12.5% SDS‒PAGE gel. The neat plasma sample was first diluted 50-fold in PBS buffer and subsequently boiled at 95 °C for 5 min. The gel was run at 90 V for the first 20 min, followed by 120 V for 80 min, and staining with Coomassie brilliant blue R-250.

### Plasma proteome sample preparation process

The M086 material was ultrasonicated in deionized water for 5 min and vortexed for 15 s, followed by 5 min of centrifugation at 14,000 RPM to remove the supernatant. The material was then resuspended in high-purity methanol to a concentration of 25 mg/mL. Then, 20 μL of the resuspended material (0.5 mg) was added to a 1.5 mL low-binding centrifuge tube and centrifuged at 14,000 RPM for 5 min to remove the supernatant. Then, 180 μL of buffer A (10 mM Tris, 150 mM KCl, 1 mM EDTA, and 0.5% CHAPS) and 20 μL of plasma were added and mixed by vertexing for 5 s and ultrasonication for 2 min. The mixture was then incubated at 25 °C and 1000 RPM in a metal bath for 15 min. Following the incubation with plasma, the material was centrifuged at 14,000 RPM for 5 min, the supernatant was removed, and 200 μL of buffer A was added to wash the material twice. The mixture was vortexed for 5 s, ultrasonicated for 2 min, and centrifuged at 14,000 RPM for 5 min each time to remove the supernatant. One hundred microlitres of a solution of DTT (20 mM) and IAA (40 mM) in NH_4_HCO_3_ (100 mM) was added to the material. The mixture was then incubated at 95 °C and 750 RPM in a metal bath for 10 min. After reduction, alkylation and cooling to room temperature, 2 μg trypsin (Promega, V5111) was added. The mixture was incubated at 37 °C overnight, followed by termination with 900 μL of 0.1% formic acid. The supernatant was collected after 5 min of centrifugation at 14,000 RPM and desalted using a 96-well HLB column (Waters, WAT058951). The desalted sample was dried by a SpeedVac^™^ vacuum concentrator. The sample was redissolved and quantified using a NanoDrop (Thermo Fisher Scientific).

### LC‒MS/MS analysis and data processing

LC‒MS/MS analysis was performed on an EASY-nLC 1200 system (Thermo Fisher Scientific) coupled to an Orbitrap Eclipse Tribrid mass spectrometer (Thermo Fisher Scientific). Five hundred nanograms of peptide was loaded onto an Acclaim^™^ PepMap^™^ 100 C18 Trap Column (Thermo Fisher Scientific) and separated by an Acclaim^™^ PepMap^™^ 100 C18 column (Thermo Fisher Scientific) using the following gradients: 5–28% solvent B (80% ACN in 0.1% FA) in 25 min, 28–45% B in 5 min, 45–95% B in 5 min and 95% B for 10 min at a flow rate of 300 nL/min. All the peptides were ionized at 2.3 kV with an ion-transfer tube temperature of 320 °C. MS1 data were acquired in a scan range of 350–1650 m/z at a resolution of 120,000 and RF lens of 40%. The normalized automatic gain control (AGC) target was set to 100% with a maximum injection time of 50 ms. The BoxCar method was set. The maximum number of multiplexed ions was set to 10. The resolution of 120,000 and RF lens of 40% were set in tSIM, the maximum injection time mode was set to Auto, and the AGC target was set to Custom. Two MS1 spectra were obtained. For MS/MS analysis, precursors with a scan range of 400–700 m/z were isolated by Quadrupole, and 60 windows of 5 m/z were established without overlap. The MS2 spectra were acquired at a scan range of 145–1450 m/z with a resolution of 30,000, HCD collision energy of 28% and RF lens of 40%. The normalized AGC target was set as a standard with the Auto maximum injection time.

Between the nano-ESI source and the Orbitrap Eclipse, the FAIMS device was set, the temperature for both the inner and outer electrode was 100 °C, the FAIMS gas was 0, and − 45 V was selected as the CV. All the LC–MS/MS data were analysed by DIA-NN version 1.8.0.

### HCC and healthy cohort sample collection and processing

The collection of HCC samples was approved by the Ethical Committee of Nanfang Hospital of Southern Medical University (ethical approval number: NFEC-2022–441) and registered on the ClinicalTrials website (NCT05719480). In brief, we retrospectively obtained blood samples from newly diagnosed HCC patients who visited Nanfang Hospital between July 2021 and August 2022 (all stages, with BCLC A stage as early-stage and the others as intermediate and advanced-stage). Venous blood was collected from patients using EDTA tubes after they were histopathologically diagnosed with HCC and before they started any antitumour treatment. The blood was centrifuged, and the upper plasma layer was collected within 3 h of collection. The tissues were then frozen at − 80 °C within 1 h. Healthy volunteers who were not diagnosed with any form of cancer or liver-related disease, such as hepatitis or cirrhosis, at the time of blood collection were recruited between June 2022 and October 2022. The blood collection and processing methods were consistent for both the HCC patients and the healthy participants, and all the subjects provided written informed consent. Participants were not necessarily fasting at the time of blood collection. A total of 56 age- and sex-matched subjects were randomly selected from both the HCC patient and healthy groups for analysis, and no significant differences were observed between the two groups based on Wilcoxon or Fisher tests.

### Biological characterization of M086

To determine the biological characteristics of M086, we utilized Fisher's exact test to conduct enrichment analysis of biological functions or features for proteins detected (in all three replicate experiments) by M086 or not detected (compared to the HPA database). First, we matched the gene column output of DIA-NN with the plasma protein group data in the HPA database. Then, we used UniProt IDs in Perseus (v1.6.15.0) to categorize proteins according to the Gene Ontology Cellular Component (CC), Molecular Function (MF), and Biological Process (BP) databases, the Kyoto Encyclopedia of Genes and Genomes (KEGG), the UniProt Keywords database, the Protein Families database (Pfam), and the Simple Modular Architecture Research Tool (SMART) database, as well as gene set enrichment analysis (GSEA). We performed Fisher’s exact test for the various annotations corresponding to proteins detected (enriched) by M086 and not detected (depleted) by M086. Finally, we constructed volcano plots (Fig. 4a) using the log2 odds and FDR values.

We employed 1D enrichment [43] analysis to compare the performance of M086, depleted plasma, and neat plasma with the HPA database in characterizing different biological functions (GOCC, GOBP, GOMF, KEGG, UniProt Keywords, GSEA, Pfam, and SMART). We used this approach to determine the 1D enrichment scores of the proteins detected via each method. The 1D annotation enrichment scores were calculated using Perseus software. The results were filtered with the following criteria: (1) the size of the annotated group (i.e., the number of protein groups with the annotation) must be greater than 10; and (2) the Benjamini–Hochberg adjusted *P* value (FDR) must be less than 2%. The final selected results are displayed in a heatmap (Fig. 4B) after Z score normalization.

### Statistics

Statistical analysis and visualization were performed using the Perseus (v1.6.15.0) and R (v4.2.0) software packages, which included MLR3, ggplot2, and Clusterprofiler. Proteomic experiments using different materials, depleted plasma and neat plasma were performed with biological replicates (n = 3) to obtain stable data. HCC cohort experiments were performed only once in random order. Functional protein annotation references are available through Perseus.

### Supplementary Information

**Additional file1: Figure S1.** M158, M086, and M909 could reduce plasma protein complexity. A The SDS‒PAGE results demonstrated that all three NPs (M158, M086, and M909 from left to right) at different concentrations (0.25 mg~2 mg/200 mL) were able to reduce the complexity of the plasma proteome. B Quantification of SDS‒PAGE data using ImageJ software showed a reduction in plasma protein complexity for all types of materials compared with that for neat plasma, especially for proteins with molecular weights (MWs) ranging from 40 to 75 kilodaltons (kDa).

**Additional file2: Figure S2.** Protein and peptide characterization of the three different NPs and the depleted and neat plasma samples. A Peptides from five different plasma methods, namely, three NP, depleted and neat plasma methods, as determined by LC‒MS/MS and DIA-NN (version 1.8.1, 1% protein and peptide FDR). The upper dashes depict the number of peptides detected in any sample; the lower dashes depict the number of peptides detected in all three replicates. The white circles show the number of peptides for each assay replicate. B CV% for precision evaluation of the five different assays for protein peptides (DIA-NN, filtering for three out of three valid values). Inner boxplots report the 25% (lower hinge), 50%, and 75% quantiles (upper hinge). Whiskers indicate observations equal to or outside the hinge ± 1.5 * interquartile range (IQR). Violin plots were generated to capture all the data points. C CV% for three biological reproducibility for different intensities of proteins. The blue lines are linear regression models. The horizontal coordinate indicates the ranking of the proteins based on their median intensities (in increasing order from left to right), and the vertical coordinate indicates the CV value of the protein. D Venn diagram showing the numbers of common proteins identified by different materials, neat plasma and exosome markers (Top 100 protein markers from the Exocarta dataset are characteristic proteins of EVs). E A deconvolution model using exosome markers (same as above) was developed to show the percentage of exosome protein intensity. F Venn diagram showing the proteins identified by M086, Depleted or Neat plasma.

**Additional file3: Figure S3.** Biological characteristics of M086 compared to depleted or neat plasma. A Heatmap of the top 50 differentially expressed proteins identified by three different methods (M086\depleted\neat plasma) via one-way ANOVA. B Results of GO analysis of differentially expressed proteins (DEPs) among the proteins identified by all three different methods (M086/depleted/neat plasma). C Results of GO analysis of DEPs between the proteins identified by M086 and depleted plasma. D The GO analysis results for proteins specifically identified by M086.

**Additional file4: Figure S4.** Protein profile characteristics of samples in the HCC study. A Heatmap of the correlation analysis results between the samples. B Kernel density map of protein intensity for each sample. Almost all the samples exhibited a normal distribution or a slight left-skewed distribution. C PLS-DA analysis showed excellent discrimination between the HCC cohort and the healthy human cohort. D S-plot revealing that numerous proteins may be associated with HCC, among which GDF15/MGLL/DTD1/RGS18/SLA2 may play an important role.

**Additional file5: Figure S5.** Protein profile characteristics of samples in the HCC study. A-B Based on LASSO coefficient path diagrams (A) and regression analysis cross-validation curves (B), 15 features were ultimately filtered out and used to construct our prediction model.C Boxplots showing the performance of the 15 protein signatures in different groups. The 25% (lower hinge), 50%, and 75% quantiles (upper hinge). * P value<0.05, ** P value<0.01, *** P value<0.001, **** P value<0.0001.D. ROC curves of top 20 plasma proteins which could distinguish patients with early-stage disease (stage A) and intermediate and advanced-stage (stage B/C) HCC.

**Additional file6: Table S1.** Physical characteristics of materials. **Table S2.** Workflow of different materials assay. **Table S3.** Performance measures for M086-based and other plasma proteomic methods. **Table S4.** Baseline of HCC Cohort. **Table S5.** Clinical information of HCC Cohort.

## Data Availability

The clinical and participant information of the HCC patients is provided in Additional file 6: Table S4 and S5. The raw mass spectrometry data are available via ProteomeXchange with the identifier PXD048186. The UniProt tool Fasta is available at https://www.uniprot.org/(retrieved 2022-10-29). All the other data are available from the corresponding authors upon reasonable request.

## Appendix 1

M158 prior to incubation with plasma

M909 prior to incubation with plasma

M086 prior to incubation with plasma

M158 after incubation with plasma

M909 after incubation with plasma

M086 after incubation with plasma

## Appendix 3

See Table 1

**Table 1:** Workflow for materials with different concentration of assay

Workflow	Steps	Wash time (min)	Centrifugation time (min)		Resuspension time (min)	Total time (min)
M158 0.25 mg	Particle wash	~ 7	~ 6	~ 8		~ 21
M158 0.50 mg	Particle wash	~ 8	~ 6	~ 16		~ 30
M158 1.0 mg	Particle wash	~ 12	~ 8	~ 14		~ 34
M158 2.0 mg	Particle wash	~ 15	~ 10	~ 18		~ 43
M909 0.25 mg	Particle wash	~ 8	~ 6	~ 12		~ 26
M909 0.50 mg	Particle wash	~ 10	~ 6	~ 20		~ 36
M909 1.0 mg	Particle wash	~ 14	~ 8	~ 24		~ 46
M909 2.0 mg	Particle wash	~ 18	~ 10	~ 30		~ 58
M086 0.25 mg	Particle wash	~ 6	~ 6	~ 2		~ 14
M086 0.50 mg	Particle wash	~ 6	~ 6	~ 3		~ 15
M086 1.0 mg	Particle wash	~ 7	~ 6	~ 4		~ 17
M086 2.0 mg	Particle wash	~ 9	~ 8	~ 5		~ 22
