# Supplementary figures and images for: The development of a novel zeolite-based assay for efficient and deep plasma proteomic profiling
Source: J Nanobiotechnology. 2024 Apr 10;22:164. doi: 10.1186/s12951-024-02404-9 (PMC11007927; doi:10.1186/s12951-024-02404-9)

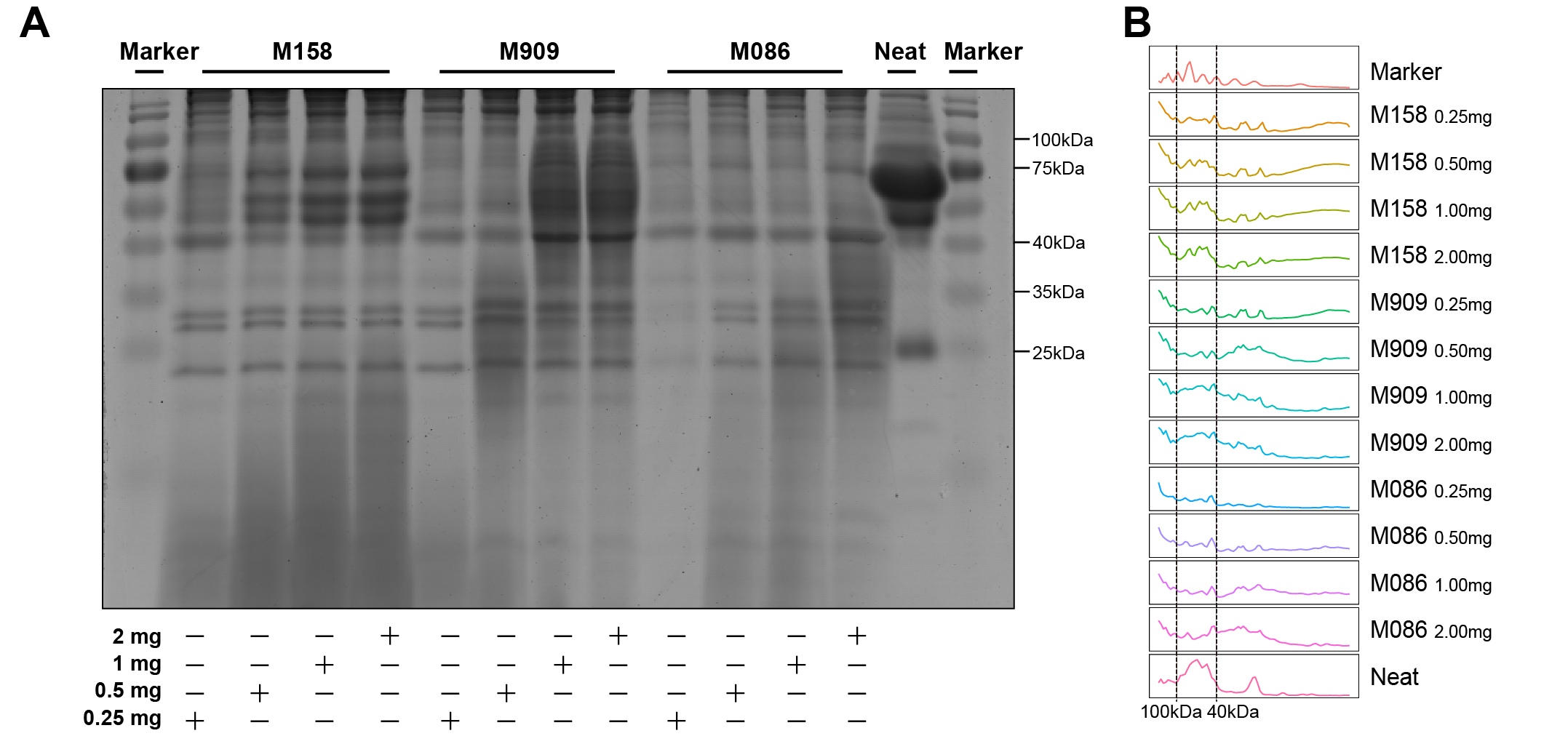

Supplement: Supplementary file 1 — Additional file1: Figure S1. M158, M086, and M909 could reduce plasma protein complexity. A The SDS‒PAGE results demonstrated that all three NPs (M158, M086, and M909 from left to right) at different concentrations (0.25 mg~2 mg/200 mL) were able to reduce the complexity of the plasma proteome. B Quantification of SDS‒PAGE data using ImageJ software showed a reduction in plasma protein complexity for all types of materials compared with that for neat plasma, especially for proteins with molecular weights (MWs) ranging from 40 to 75 kilodaltons (kDa). [file 12951_2024_2404_MOESM1_ESM.jpg]

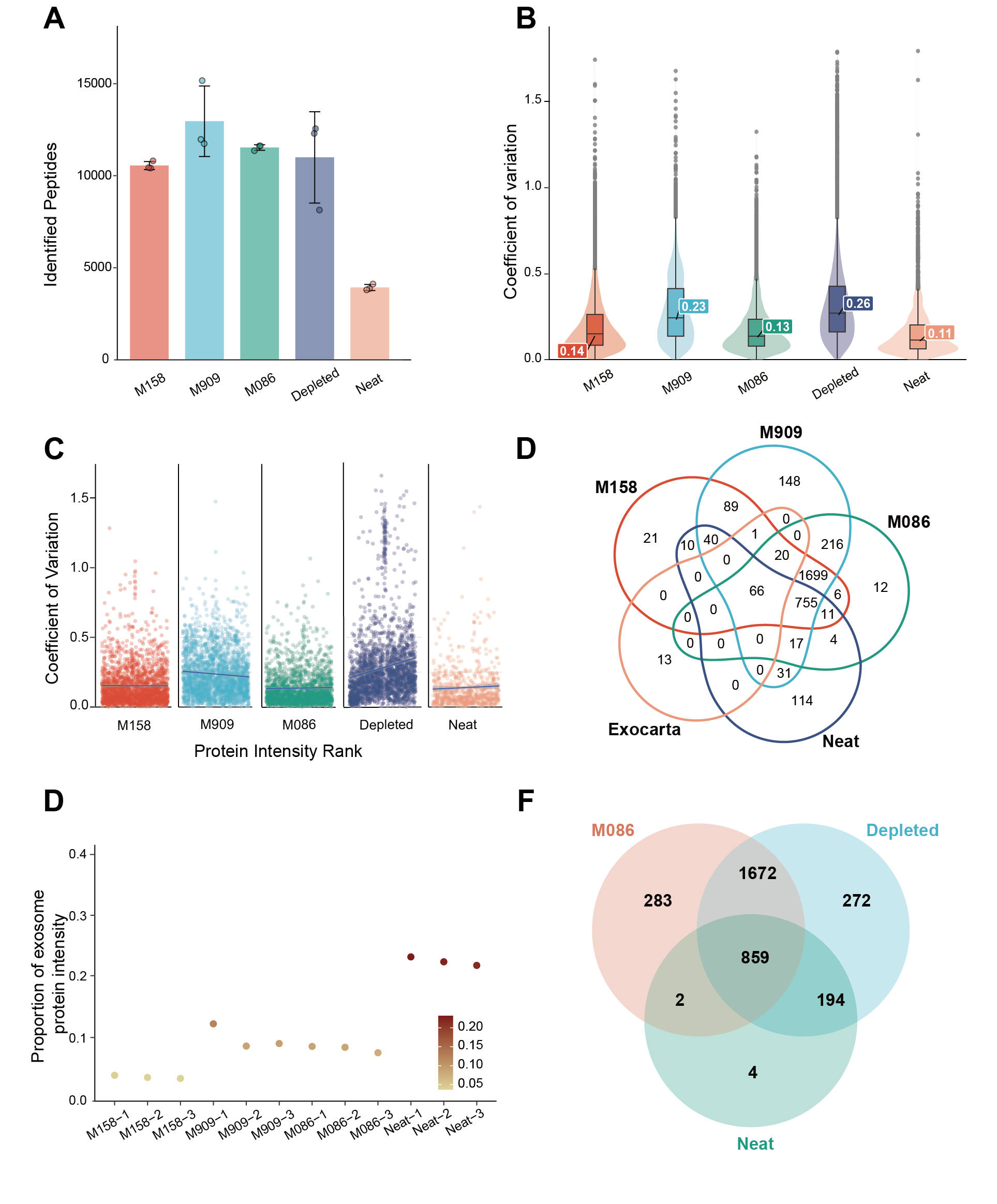

Supplement: Supplementary file 2 — Additional file2: Figure S2. Protein and peptide characterization of the three different NPs and the depleted and neat plasma samples. A Peptides from five different plasma methods, namely, three NP, depleted and neat plasma methods, as determined by LC‒MS/MS and DIA-NN (version 1.8.1, 1% protein and peptide FDR). The upper dashes depict the number of peptides detected in any sample; the lower dashes depict the number of peptides detected in all three replicates. The white circles show the number of peptides for each assay replicate. B CV% for precision evaluation of the five different assays for protein peptides (DIA-NN, filtering for three out of three valid values). Inner boxplots report the 25% (lower hinge), 50%, and 75% quantiles (upper hinge). Whiskers indicate observations equal to or outside the hinge ± 1.5 * interquartile range (IQR). Violin plots were generated to capture all the data points. C CV% for three biological reproducibility for different intensities of proteins. The blue lines are linear regression models. The horizontal coordinate indicates the ranking of the proteins based on their median intensities (in increasing order from left to right), and the vertical coordinate indicates the CV value of the protein. D Venn diagram showing the numbers of common proteins identified by different materials, neat plasma and exosome markers (Top 100 protein markers from the Exocarta dataset are characteristic proteins of EVs). E A deconvolution model using exosome markers (same as above) was developed to show the percentage of exosome protein intensity. F Venn diagram showing the proteins identified by M086, Depleted or Neat plasma. [file 12951_2024_2404_MOESM2_ESM.jpg]

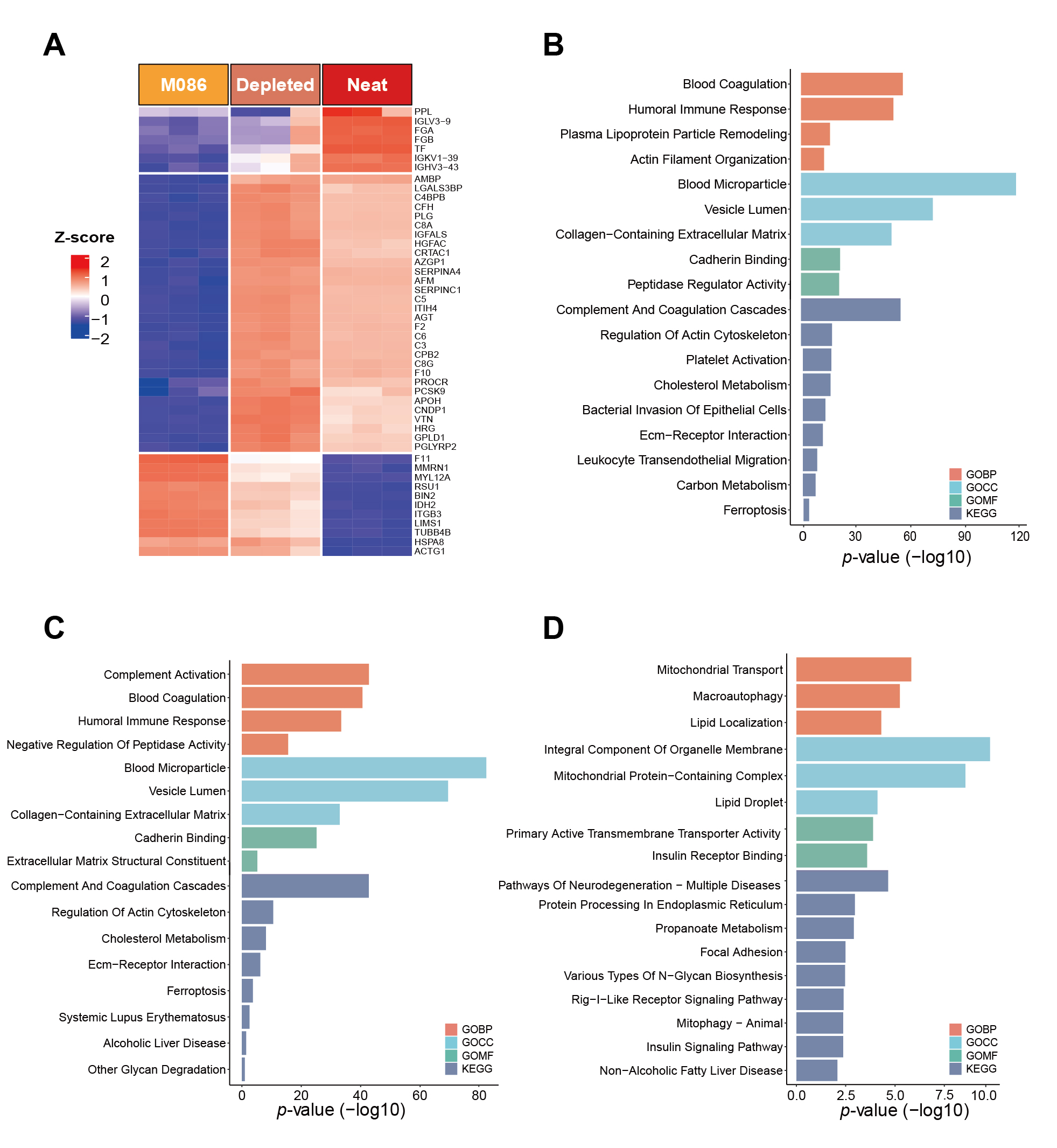

Supplement: Supplementary file 3 — Additional file3: Figure S3. Biological characteristics of M086 compared to depleted or neat plasma. A Heatmap of the top 50 differentially expressed proteins identified by three different methods (M086\depleted\neat plasma) via one-way ANOVA. B Results of GO analysis of differentially expressed proteins (DEPs) among the proteins identified by all three different methods (M086/depleted/neat plasma). C Results of GO analysis of DEPs between the proteins identified by M086 and depleted plasma. D The GO analysis results for proteins specifically identified by M086. [file 12951_2024_2404_MOESM3_ESM.jpg]

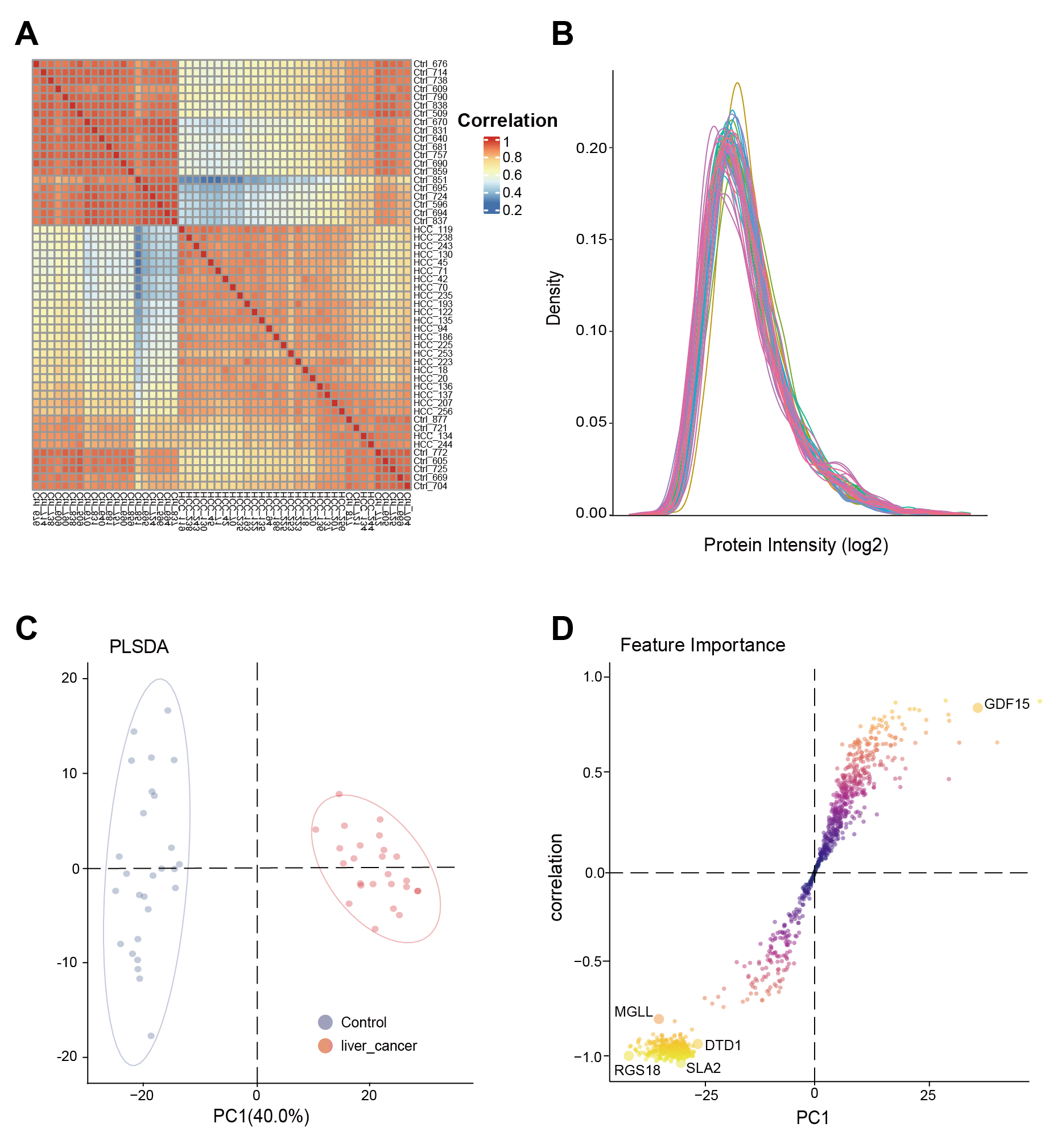

Supplement: Supplementary file 4 — Additional file4: Figure S4. Protein profile characteristics of samples in the HCC study. A Heatmap of the correlation analysis results between the samples. B Kernel density map of protein intensity for each sample. Almost all the samples exhibited a normal distribution or a slight left-skewed distribution. C PLS-DA analysis showed excellent discrimination between the HCC cohort and the healthy human cohort. D S-plot revealing that numerous proteins may be associated with HCC, among which GDF15/MGLL/DTD1/RGS18/SLA2 may play an important role. [file 12951_2024_2404_MOESM4_ESM.jpg]

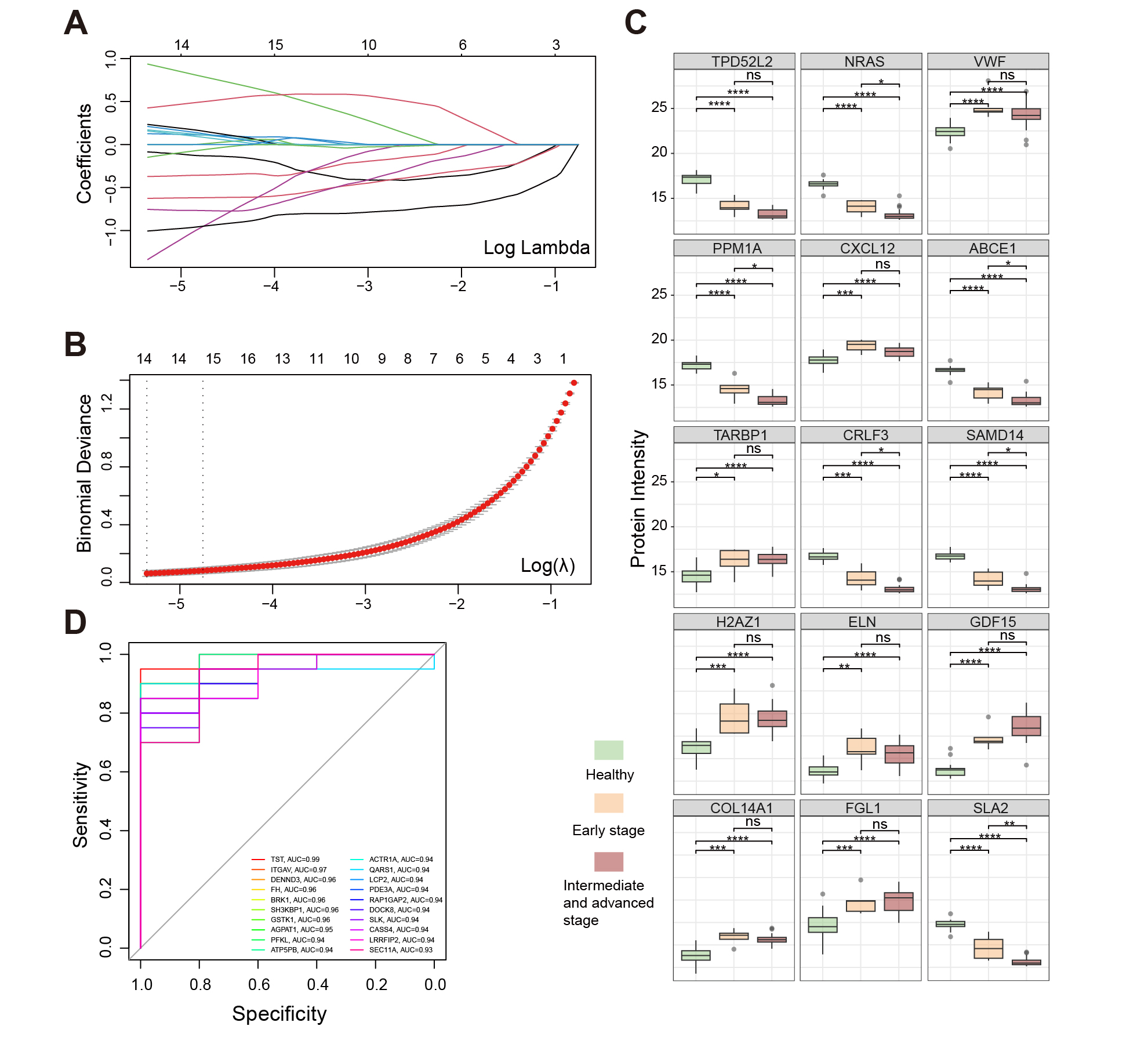

Supplement: Supplementary file 5 — Additional file5: Figure S5. Protein profile characteristics of samples in the HCC study. A-B Based on LASSO coefficient path diagrams (A) and regression analysis cross-validation curves (B), 15 features were ultimately filtered out and used to construct our prediction model.C Boxplots showing the performance of the 15 protein signatures in different groups. The 25% (lower hinge), 50%, and 75% quantiles (upper hinge). * P value<0.05, ** P value<0.01, *** P value<0.001, **** P value<0.0001.D. ROC curves of top 20 plasma proteins which could distinguish patients with early-stage disease (stage A) and intermediate and advanced-stage (stage B/C) HCC. [file 12951_2024_2404_MOESM5_ESM.jpg]
