# Supplementary material for: The development of a novel zeolite-based assay for efficient and deep plasma proteomic profiling
Source: J Nanobiotechnology. 2024 Apr 10;22:164. doi: 10.1186/s12951-024-02404-9 (PMC11007927; doi:10.1186/s12951-024-02404-9)
Supplement: Supplementary file 6 — Additional file6: Table S1. Physical characteristics of materials. Table S2. Workflow of different materials assay. Table S3. Performance measures for M086-based and other plasma proteomic methods. Table S4. Baseline of HCC Cohort. Table S5. Clinical information of HCC Cohort. [file 12951_2024_2404_MOESM6_ESM.docx]

**Additional Table 1 Physical characteristics of materials**

|  | **M158** | **M909** | **M086** |
| --- | --- | --- | --- |
| **Type** | CHA | FAU | NaY |
| **DLS (nm)** | 370.7±25.8 | 244.3±29.3 | 488.6±13.4 |
| **Si/Al (ratio)** | 2 | 2 | 2.6 |
| **Products** | Synthesized in own laboratory | Synthesized in own laboratory | Customized from Nanjing/Jiangsu XFNANO Materials Tech Co.,Ltd |
| ***Number of Proteins Identified** | 2732 | 3099 | 2822 |
| *Number of proteins identified in at least one of the three biological repeats. | | | |

**Additional Table 2 Workflow of different materials assay**

| **Workflow** | **Steps** | **Time (h)** | **Total  Time (h)** | **Products** | **Brand** | **Part Number** |
| --- | --- | --- | --- | --- | --- | --- |
| **M158**  **3 samples/batch** | Particle wash | 1.5(0.5*3) | ~18.5 | \ | \ | \ |
|  | Incubation | 1.5 |  | \ | \ | \ |
|  | Reduction, alkylation | 0.6 |  | DTT and IAA | Solarbio | D8220-5g/I8010-5g |
|  | Protein Digestion | 6 |  | Trypsin | Promega | V5111 |
|  | Desalt | 1.5 |  | HLB | Water | WAT058951 |
|  | Dehydration | 4 |  | \ | \ | \ |
|  | Reconstitution | 0.5 |  | \ | \ | \ |
|  | LC‒MS | 2.85(0.95*3) |  | Thermo Scientific Orbitrap Eclipse | Thermo Scientific | \ |
| **M909 3 samples/batch** | Particle wash | 1.8(0.6*3) | ~19.2 | \ | \ | \ |
|  | Incubation | 1.8 |  | \ | \ | \ |
|  | Reduction, alkylation | 0.7 |  | DTT and IAA | Solarbio | D8220-5g/I8010-5g |
|  | Protein Digestion | 6 |  | Trypsin | Promega | V5111 |
|  | Desalt | 1.5 |  | HLB | Water | WAT058951 |
|  | Dehydration | 4 |  | \ | \ | \ |
|  | Reconstitution | 0.5 |  | \ | \ | \ |
|  | LC‒MS | 2.85(0.95*3) |  | Thermo Scientific Orbitrap Eclipse | Thermo Scientific | \ |
| **M086 3 samples/batch** | Particle wash | 0.75(0.25*3) | ~16.9 | \ | \ | \ |
|  | Incubation | 1 |  | \ | \ | \ |
|  | Reduction, alkylation | 0.3 |  | DTT and IAA | Solarbio | D8220-5g/I8010-5g |
|  | Protein Digestion | 6 |  | Trypsin | Promega | V5111 |
|  | Desalt | 1.5 |  | HLB | Water | WAT058951 |
|  | Dehydration | 4 |  | \ | \ | \ |
|  | Reconstitution | 0.5 |  | \ | \ | \ |
|  | LC‒MS | 2.85(0.95*3) |  | Thermo Scientific Orbitrap Eclipse | Thermo Scientific | \ |

**Additional Table 3 Performance measures for M086-based and other plasma proteomic methods**

| **Assay** | **Protein Groups** | **Median CV(%)** | **Detection Limit** | **Sample Volume(μL)** | **Total Assay Time (h)** | **Cost per Sample** | **Reference** |  |
| --- | --- | --- | --- | --- | --- | --- | --- | --- |
| **Immunoaffinity Depletion combined with LC‒MS** | ~1000 | ~10% | mg/L | 100 | ~48 hrs | High | Keshishian, Burgess et al. 2017  PMID: 28749931 |  |
|  |  |  |  |  |  |  |  |  |
|  |  |  |  |  |  |  |  |  |
|  |  |  |  |  |  |  |  |  |
|  |  |  |  |  |  |  |  |  |
|  |  |  |  |  |  |  |  |  |
|  |  |  |  |  |  |  |  |  |
|  |  |  |  |  |  |  |  |  |
| **Multinanoparticle Plasma Proteomic Assay** | ~1650 | <20% | ng/L | 20 | 7 hrs sample prep  + 2.5 hrs MS | High | Ferdosi, Tangeysh et al. 2022  PMID: 35275789 |  |
|  |  |  |  |  |  |  |  |  |
|  |  |  |  |  |  |  |  |  |
|  |  |  |  |  |  |  |  |  |
|  |  |  |  |  |  |  |  |  |
|  |  |  |  |  |  |  |  |  |
|  |  |  |  |  |  |  |  |  |
| **M086-based Plasma Proteomic Assay** | ~4000 | <20% | ng/L | 25 | 10.5 hrs sample prep + 1 hrs MS | Low | / |  |
|  |  |  |  |  |  |  |  |  |
|  |  |  |  |  |  |  |  |  |
|  |  |  |  |  |  |  |  |  |
|  |  |  |  |  |  |  |  |  |
|  |  |  |  |  |  |  |  |  |
|  |  |  |  |  |  |  |  |  |
| **Aptamer-based Plasma Proteomic Assay** | ~7000 | ~5% | ng/L | 55 | ~48 hrs | High | Candia, Daya et al. 2022  PMID: 36229504 |  |
|  |  |  |  |  |  |  |  |  |
|  |  |  |  |  |  |  |  |  |
|  |  |  |  |  |  |  |  |  |
|  |  |  |  |  |  |  |  |  |
|  |  |  |  |  |  |  |  |  |
|  |  |  |  |  |  |  |  |  |
| **PEA-based Plasma Proteomic Assay** | ~3000 | ~20% | ng/L | 80 | <36 hrs | High | Zhong, Edfors et al. 2021  PMID: 33941778 |  |
|  |  |  |  |  |  |  |  |  |
|  |  |  |  |  |  |  |  |  |
|  |  |  |  |  |  |  |  |  |
|  |  |  |  |  |  |  |  |  |
|  |  |  |  |  |  |  |  |  |
|  |  |  |  |  |  |  |  |  |

**Additional Table 4 Baseline of HCC Cohort**

|  | **Control** | | **HCC** | ***p*** | |
| --- | --- | --- | --- | --- | --- |
|  | **（n=27）** | | **（n=25）** |  |  |
| **Age, years** | | 56.15 (9.80) | 52.04 (13.31) | 0.209 | |
| *mean (SD)* | |  |  |  |  |
| **Sex** | | | | | |
| *Male* | 16 (59.3) | | 19 (76.0) | 0.322 | |
| *Female* | 11 (40.7) | | 6 (24.0) |  |  |
|  |  |  |  |  |  |

| **Additional Table 5 Clinical information of HCC Cohort** | | | | | | | |
| --- | --- | --- | --- | --- | --- | --- | --- |
| Sample ID | Group | Sex | Age | *Stage | HBV infection | Cirrhosis | Pathology |
| 256 | HCC | male | 34 | C | No | No | hepatocellular carcinoma |
| 94 | HCC | male | 34 | C | No | No | hepatocellular carcinoma |
| 253 | HCC | male | 69 | C | Yes | Yes | hepatocellular carcinoma |
| 243 | HCC | male | 74 | C | Yes | Yes | hepatocellular carcinoma |
| 207 | HCC | female | 25 | A | Yes | Yes | hepatocellular carcinoma |
| 225 | HCC | male | 39 | C | Yes | No | hepatocellular carcinoma |
| 134 | HCC | male | 56 | A | Yes | Yes | hepatocellular carcinoma |
| 186 | HCC | male | 63 | 0 | Yes | Yes | hepatocellular carcinoma |
| 20 | HCC | male | 52 | C | Yes | Yes | hepatocellular carcinoma |
| 119 | HCC | male | 64 | C | Yes | Yes | hepatocellular carcinoma |
| 223 | HCC | female | 61 | C | Yes | Yes | hepatocellular carcinoma |
| 136 | HCC | male | 49 | C | Yes | Yes | hepatocellular carcinoma |
| 18 | HCC | female | 25 | A | No | No | hepatocellular carcinoma |
| 122 | HCC | male | 62 | C | Yes | Yes | hepatocellular carcinoma |
| 135 | HCC | male | 39 | C | Yes | Yes | hepatocellular carcinoma |
| 193 | HCC | female | 61 | C | Yes | Yes | hepatocellular carcinoma |
| 70 | HCC | male | 55 | C | Yes | Yes | hepatocellular carcinoma |
| 130 | HCC | male | 47 | C | Yes | Yes | hepatocellular carcinoma |
| 137 | HCC | male | 59 | C | Yes | Yes | hepatocellular carcinoma |
| 244 | HCC | male | 62 | A | Yes | Yes | hepatocellular carcinoma |
| 42 | HCC | female | 48 | C | Yes | Yes | hepatocellular carcinoma |
| 45 | HCC | female | 66 | C | Yes | Yes | hepatocellular carcinoma |
| 235 | HCC | male | 59 | B | Yes | Yes | hepatocellular carcinoma |
| 71 | HCC | male | 52 | B | Yes | Yes | hepatocellular carcinoma |
| 238 | HCC | male | 46 | C | Yes | Yes | hepatocellular carcinoma |
| 596 | Control | male | 52 | / | No | No | / |
| 609 | Control | male | 59 | / | No | No | / |
| 640 | Control | male | 72 | / | No | No | / |
| 670 | Control | female | 54 | / | No | No | / |
| 682 | Control | male | 70 | / | No | No | / |
| 694 | Control | female | 52 | / | No | No | / |
| 695 | Control | male | 60 | / | No | No | / |
| 714 | Control | male | 54 | / | No | No | / |
| 721 | Control | male | 59 | / | No | No | / |
| 738 | Control | female | 62 | / | No | No | / |
| 757 | Control | female | 65 | / | No | No | / |
| 790 | Control | female | 51 | / | No | No | / |
| 837 | Control | male | 66 | / | No | No | / |
| 838 | Control | female | 67 | / | No | No | / |
| 851 | Control | female | 52 | / | No | No | / |
| 509 | Control | male | 55 | / | No | No | / |
| 605 | Control | female | 72 | / | No | No | / |
| 669 | Control | male | 46 | / | No | No | / |
| 676 | Control | male | 49 | / | No | No | / |
| 690 | Control | female | 57 | / | No | No | / |
| 704 | Control | male | 55 | / | No | No | / |
| 724 | Control | female | 63 | / | No | No | / |
| 725 | Control | male | 52 | / | No | No | / |
| 772 | Control | male | 54 | / | No | No | / |
| 831 | Control | male | 53 | / | No | No | / |
| 859 | Control | male | 34 | / | No | No | / |
| 877 | Control | female | 31 | / | No | No | / |
| * Barcelona Clinic Liver Cancer (BCLC) stage. | | | | | | | |
